# The upregulation of POLR3G correlates with increased malignancy of bladder urothelium

**DOI:** 10.1186/s40001-024-01980-8

**Published:** 2024-07-22

**Authors:** Xianhui Liu, Lin Zhu, Diancheng Li, Xiao Chen

**Affiliations:** 1 Department of Urology, Beijing Jishuitan Hospital Affiliated to Capital Medical University, No. 31 Xinjiekou East Street, Xicheng District, Beijing, 100035 China; 2 Department of Plastic Surgery, Beijing Chaoyang Hospital Affiliated to Capital Medical University, Beijing, China; 3 Department of Ultrasound, Peking University People’s Hospital, Beijing, China; 4 Department of Urology, Shaanxi Provincial People’s Hospital, Shaanxi, China

## Abstract

Bladder cancer remains a significant health challenge due to its high recurrence and progression rates. This study aims to evaluate the role of POLR3G in the development and progression of bladder cancer and the potential of POLR3G to serve as a novel therapeutic target. We constructed a bladder cancer model in Wistar rats by administering N-butyl-N-(4-hydroxybutyl) nitrosamine (BBN), which successfully induced a transition from normal mucosa to hyperplasia and ultimately to urothelial carcinoma. We observed a progressive upregulation of POLR3G expression during the bladder cancer development and progression. To investigate the functional role of POLR3G, we performed functional experiments in bladder cancer cell lines. The results demonstrated that knocking down POLR3G significantly inhibited cell proliferation, migration, and invasion. We further conducted RNA sequencing on POLR3G-knockdown bladder cancer cells, and Metascape was employed to perform the functional enrichment analysis of the differentially expressed genes (DEGs). Enrichment analysis revealed the enrichment of DEGs in the RNA polymerase and apoptotic cleavage of cellular proteins pathways, as well as their involvement in the Wnt and MAPK signaling pathways. The downregulation of Wnt pathway-related proteins such as Wnt5a/b, DVL2, LRP-6, and phosphorylated LRP-6 upon POLR3G knockdown was further confirmed by Western blotting, indicating that POLR3G might influence bladder cancer behavior through the Wnt signaling pathway. Our findings suggest that POLR3G plays a crucial role in bladder cancer progression and could serve as a potential therapeutic target. Future studies should focus on the detailed mechanisms by which POLR3G regulates these signaling pathways and its potential as a biomarker for early detection and prognosis of bladder cancer.

## Introduction

Bladder cancer stands as a significant global health concern [1]. According to the Global Cancer Observatory (GLOBOCAN) data from 2022, bladder cancer is the sixth most commonly diagnosed cancer in the male population worldwide, and it is the eleventh when both genders are considered. There were approximately 614,298 new cases of bladder cancer and 220,596 deaths globally [2]. The management of bladder cancer necessitates a multidisciplinary approach based on the patient’s tumor grade, tumor stage, overall health, and individualized treatment options. Surgery plays a pivotal role in the treatment of bladder cancer. Transurethral resection of bladder tumor (TURBT) is often the first step in managing non-muscle invasive bladder cancer (NMIBC), followed by intravesical chemotherapy or intravesical Bacillus Calmette–Guérin (BCG) therapy [3]. Meanwhile, patients with muscle-invasive bladder cancer (MIBC) often require radical cystectomy and urinary diversion [4]. Platinum-based chemotherapy is an integral part of bladder cancer treatment, which can be administered as neoadjuvant therapy to downstage the tumor or adjuvant therapy to control recurrence and reduce the risk of metastasis [5]. While patients with advanced bladder cancer have poor prognoses due to limited treatment options and a high rate of recurrence [4, 6]. Platinum-based chemotherapy has been the first-line treatment of advanced bladder cancer since the late 1980s [7]. Over the past decade, the advent of immune checkpoint inhibitors (ICIs) and antibody–drug conjugates (ADCs) has revolutionized the treatment landscape of bladder cancer. The landmark phase III trials, including KEYNOTE-045 [8], CheckMate 275 [9], and IMvigor211 [10], have provided robust evidence supporting the use of ICIs as a standard therapeutic option in the management of advanced bladder cancer, with an overall objective response rate (ORR) of 21.1%, 19.6%, and 15%. ADCs have demonstrated efficacy against specific molecular targets overexpressed in tumor cells, such as Nectin-4 and Trop-2 [11, 12]. Enfortumab vedotin [11] demonstrated an ORR of 44%, and sacituzumab govitecan [12] demonstrated an ORR of 27% in patients with locally advanced or metastatic urothelial carcinoma who were previously treated with chemotherapy and ICIs.

Despite advances in diagnostic and therapeutic strategies, the prognosis for advanced bladder cancer remains poor, necessitating the exploration of novel molecular targets and therapeutic approaches. In our previous study, we found POLR3G was up-regulated in bladder cancer, and higher expression of POLR3G was associated with more advanced tumor stage and poorer prognosis [13]. In this study, we aim to evaluate the dynamic expression of POLR3G during the development of bladder cancer in animal models and the potential role of POLR3G to serve as a novel therapeutic target.

## Materials and methods

### Animal models

Female Wistar rats and N-butyl-N-(4-hydroxybutyl) nitrosamine (BBN, TCI), a chemical carcinogen, was used to generate the bladder cancer model. A total of 35 (15 of control group and 20 of experimental group) female Wistar rats of 5 weeks were purchased from Charles River, and were housed in a specific-pathogen free facility at 3–5 per cage with 12 h light/dark cycles and with ad libitum access to food. Rats in control group were fed with freely available drinking water. Rats in experimental group for bladder tumor models were fed with water added with 0.05% BBN in dark bottles. Each 3 rats in control group were scheduled for ultrasound checking and haematoxylin and eosin (HE) staining of bladders in week 0, week 5, week 10, week 15, and week 20, and each 5 rats in experimental group were scheduled for ultrasound and HE staining of bladders in week 5, week 10, week 15, and week 20. Rats were anesthetized with isoflurane gas during ultrasound checking for bladder tumors, and were euthanized by asphyxiation with carbon dioxide before the bladders were harvested for HE staining and western blotting assays. All rats were closely monitored for any distress or pain throughout the study period and were monitored to determine a humane endpoint (> 20% body weight loss, physical inactivity or signs of severe toxicity such as infections, bleeding, or diarrhea) was reached.

### Western blotting analysis

Total protein from bladder tissues was extracted using RIPA lysis and extraction buffer (Solarbio) and measured using a BCA kit (Solarbio). A total of 20 mg protein from each sample was separated using 10% separating gels and transferred to polyvinylidene fluoride membranes (Solarbio). Proteins were detected using a Fluorescence Imaging System (Sagecreation). Antibody information is summarized in Supplementary Table S1.

### Cell lines and cell culture

Human bladder cancer cell lines, T24 and BIU87, were purchased from National Infrastructure of Cell Line Resource (Beijing, China). Cell lines were maintained in Roswell Park Memorial Institute (RPMI) 1640 medium (Gibco) supplemented with 10% fetal bovine serum (Gibco) and 1% penicillin/streptomycin (Gibco). All cells were maintained in a humidified atmosphere with 5% CO2 at 37 °C.

### Vector construction and cell infection

Synthesis of small interfering RNA (siRNA) targeting POLR3G and negative control siRNAs was completed by GenePharma. The siRNA construct with the greatest POLR3G silencing efficiency and a negative control (siNC) were cloned into lentiviral vectors to create stable POLR3G-knockdown cells. The siRNA transfection or viral infection was completed according to the manufacturer's instructions. The efficiency of siRNA transfection or viral infection was verified by qRT-PCR or Western blotting. The siRNA, short hairpin RNA (shRNA), and corresponding control sequences are summarized in Supplementary Table S2.

### RNA extraction and qRT-PCR

Total RNA was extracted from cells using the RNA simple Total RNA Kit (Tiangen). FastQuant RT Kit (Tiangen) was used for cDNA synthesis. The quantitative real time polymerase chain reactions (qRT-PCR) were performed using KAPA SYBR FAST Universal q-PCR Kit (KAPA). The relative mRNA levels of genes were calculated using cycle threshold (CT) methods, and β-actin was used as an endogenous control. Three replicate samples were studied for detection of mRNA expression. The primers sequences are summarized in Supplementary Table S2.

### CCK-8 assays

The effect of POLR3G on cell proliferation was evaluated using the cell counting kit-8 (CCK-8) assay (Dojindo). Briefly, 1500 cells in 150 mL of medium were seeded onto 96-well plates. The absorbance of each well at 450 nm was measured at six different time points. Prior to all absorbance measurements, the medium in each well was replaced with 100 mL of complete medium supplemented with 10% CCK-8 solution, and the cells were incubated for 2 h.

### Cell migration and invasion assays

Cell migration and invasion were evaluated using Transwell invasion assays with or without Matrigel. To assess the effect of POLR3G on cell migration and invasion, 4*10^4^ cells were plated into the upper chamber of a 24-well Transwell or Matrigel chamber with 8-mm pores (Corning). For cell migration assays, T24 and BIU87 cells were incubated for 24 h prior to the assay. For cell invasion assays, T24 and BIU87 cells were incubated for 48 h, respectively. Migrating and invading cells were fixed with 4% paraformaldehyde before 0.5% crystal violet staining. The remaining cells were recorded photographically and counted in different fields triply.

### RNA sequencing and bioinformatic analysis

The transcriptomic analysis was performed using the MGI high-throughput sequencing platform. Differentially expressed genes (DEGs) were selected using the DEGSeq package [14]. An FDR cut-off of 0.05 and absolute fold change > 1.5 was used to select statistically significantly DEGs. Metascape was used to enrich genes for GO biological processes, KEGG Pathway and Reactome Gene Sets [15].

## Results

### Establishment of the BBN-induced bladder *cancer* models

**Fig. 1:**
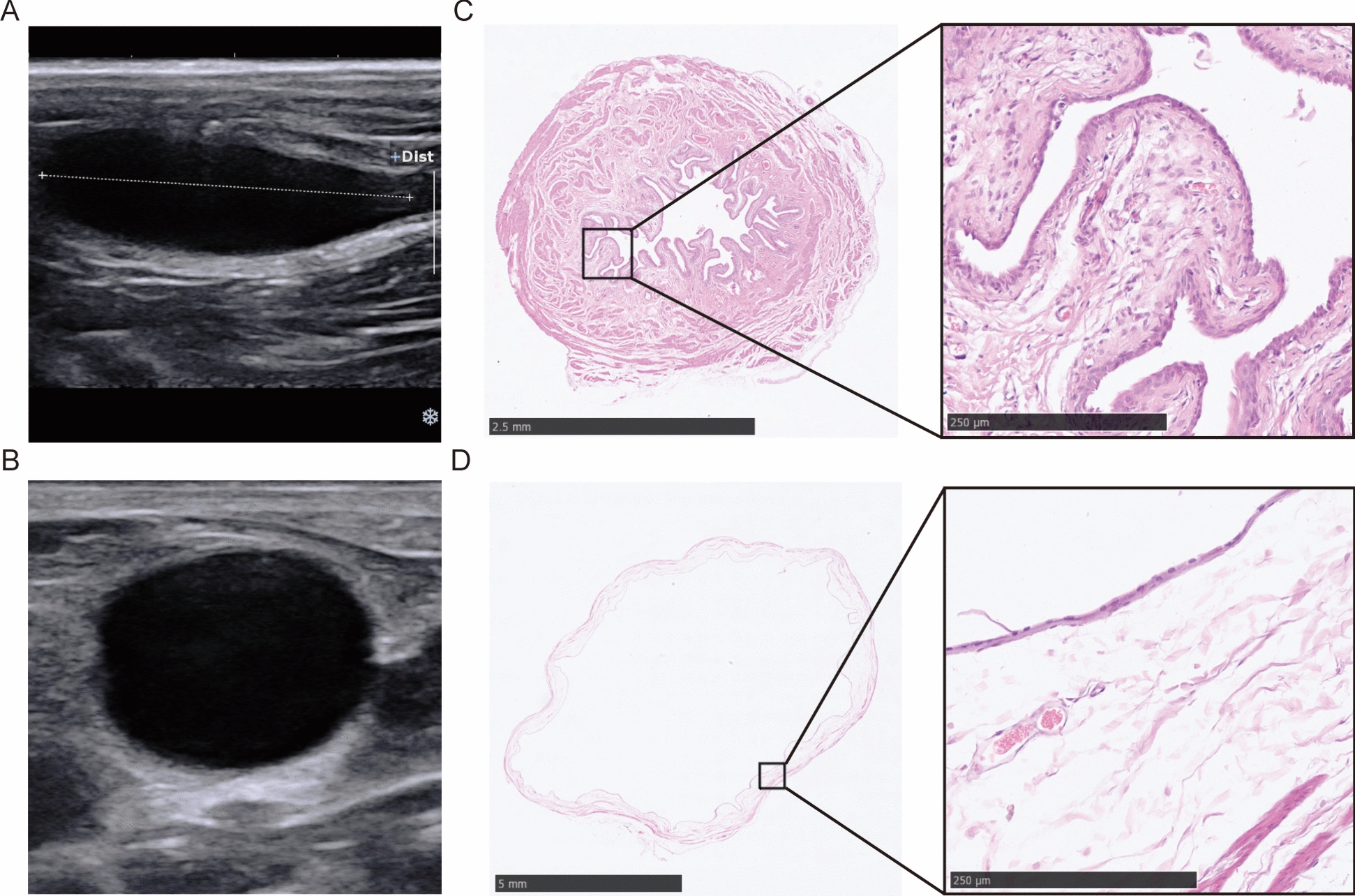
The images of ultrasonography and HE staining of normal rat bladders

The images of ultrasonography and HE staining of normal rat bladders are shown in Fig. 1. The normal rat bladder appeared teardrop-shaped under ultrasound (Fig. 1A, B). HE staining showed that the urothelium protrude into the bladder cavity in a papillary manner in the unfilled state (Fig. 1C), while in the filling state, the urinary epithelium appeared as a single layer of urinary epithelial cells (Fig. 1D).

**Fig. 2:**
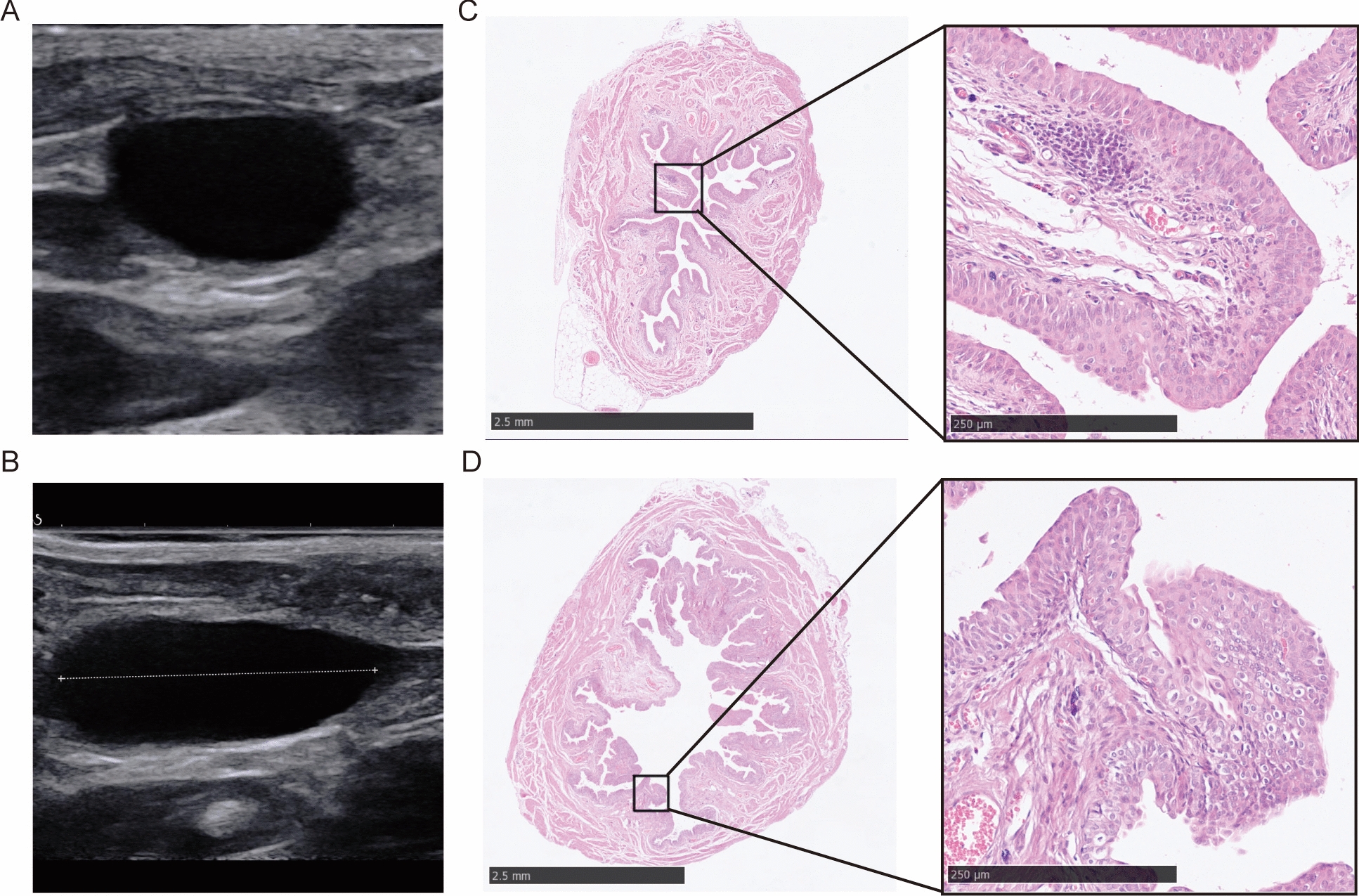
The images of ultrasonography and HE staining of rat bladders after BBN induction for 5–10 weeks

After 5 to 10 weeks of BBN induction, no abnormal echo was found by ultrasonography (Fig. 2A, B), while HE staining showed significant hyperplasia, with an increase from 1 layer of epithelial cells to multiple layers, accompanied with loss of polarity features and lymphocytic infiltration (Fig. 2C, D).

**Fig. 3:**
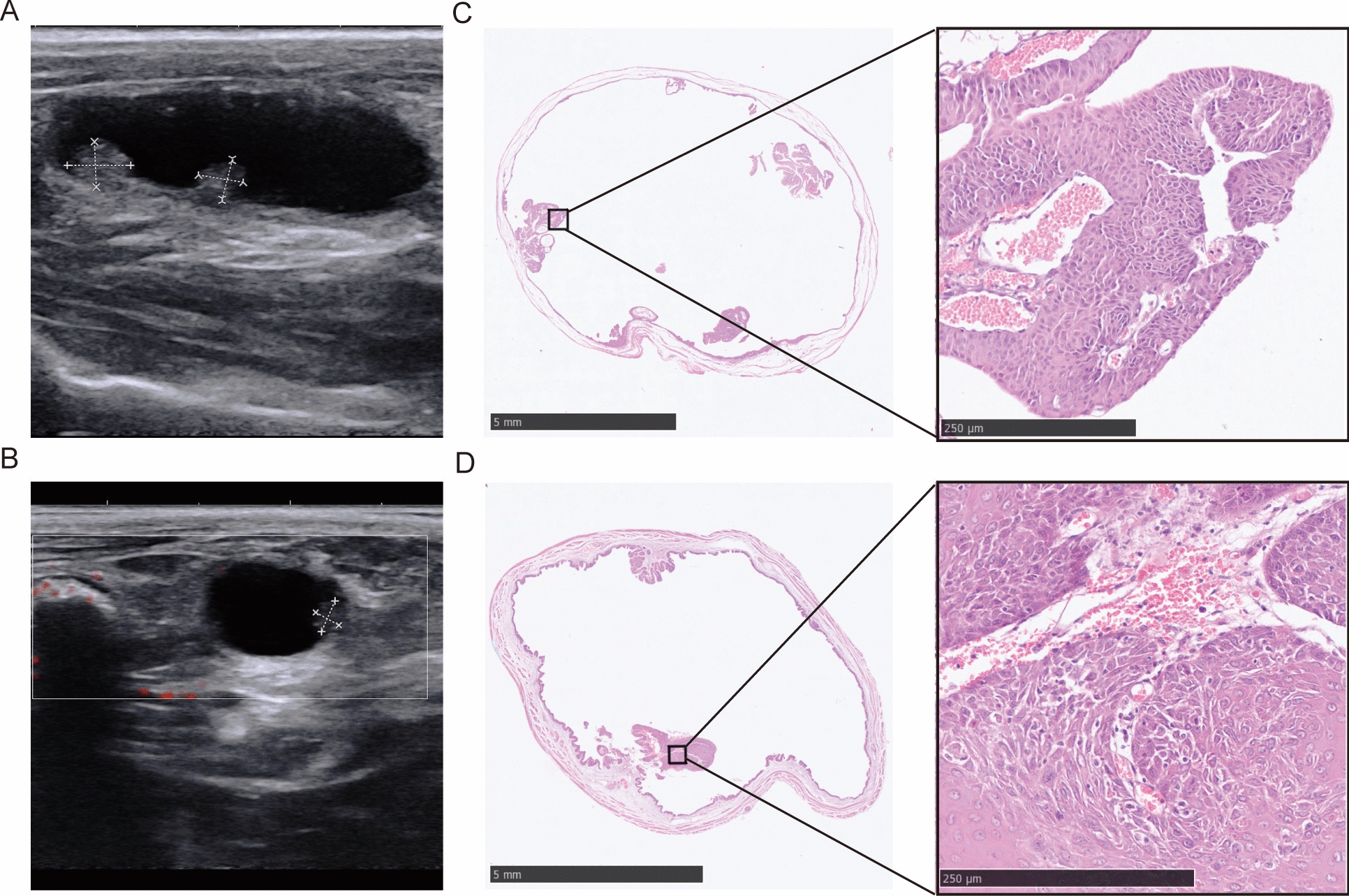
The images of ultrasonography and HE staining of rat bladders after BBN induction for 15–20 weeks

After 15 to 20 weeks of BBN induction, ultrasound revealed hyperechoic masses protruding into the bladder cavity (Fig. 3A, B), which were confirmed as urothelial carcinomas by HE staining. (Fig. 3C, D).

### POLR3G expression is positively correlated with tumor progression

**Fig. 4:**
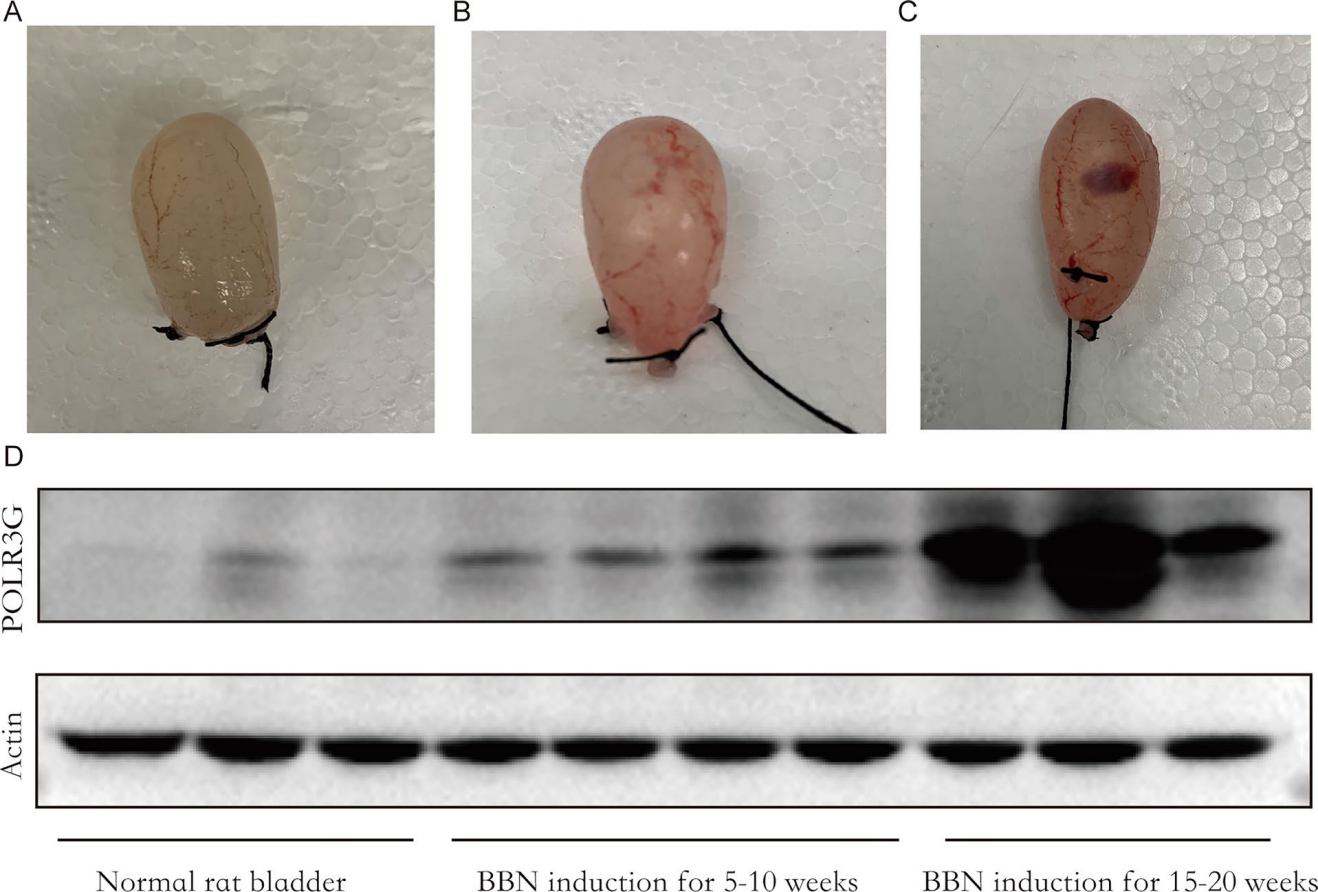
The dynamic change of POLR3G expression during the development of BBN-induced bladder cancer

As showed in Figs. 1–3, microscopic lesions in rats start as hyperplasia, evolving into papillary carcinomas after BBN induction. We examined the dynamic change of POLR3G expression during the development of BBN-induced bladder cancer. Results showed that POLR3G was lowly expressed in the normal urothelium of rats, while its expression was significantly up-regulated during the development of BBN-induced bladder cancer (Fig. 4).

### Knockdown of POLR3G decreases cell proliferation, migration and invasion in bladder *cancer* cells

**Fig. 5:**
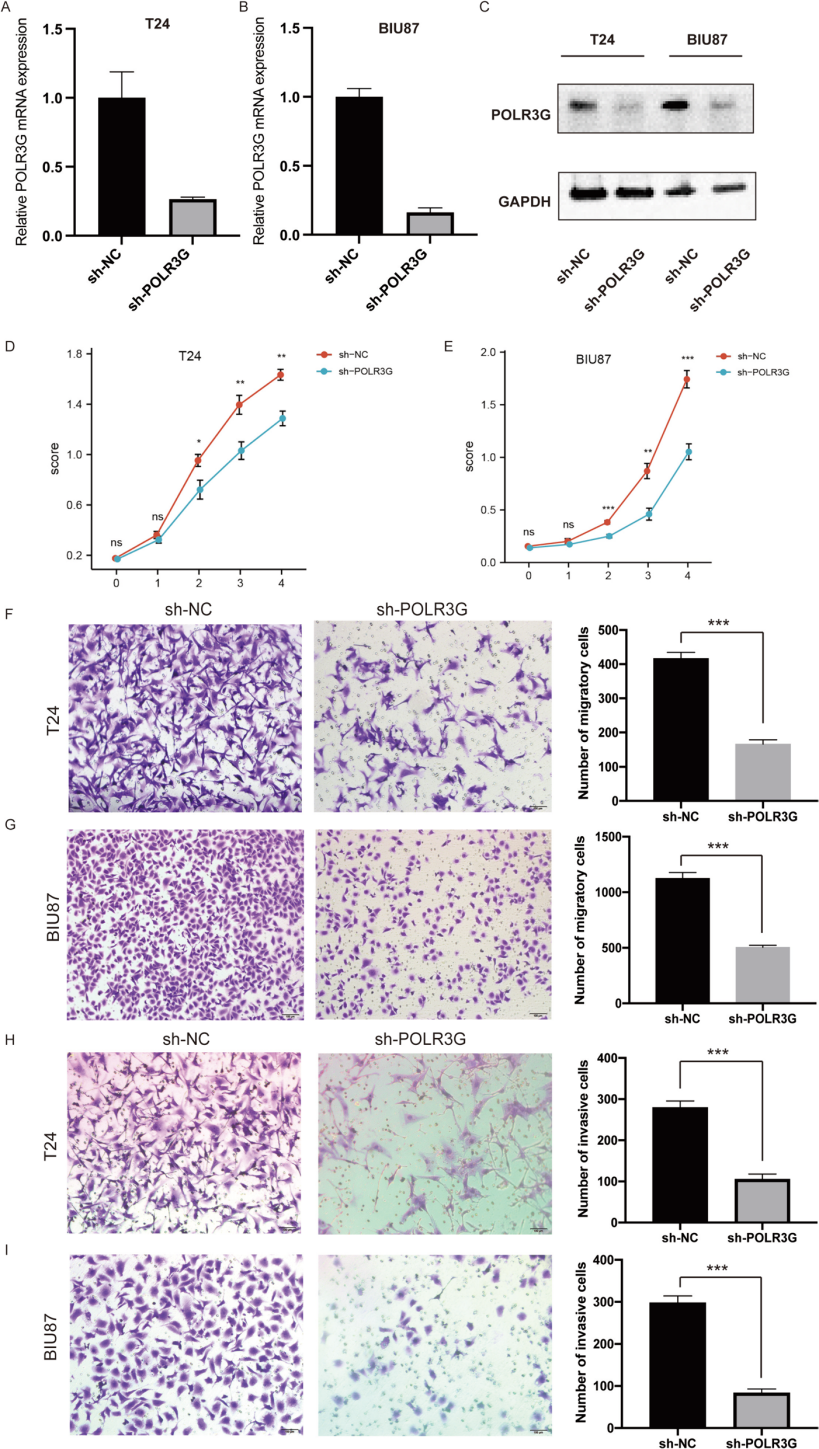
The knockdown of POLR3G inhibited the proliferation, migration, and invasion capabilities of bladder cancer cells

To investigate the molecular function of POLR3G in bladder cancer cells, we knocked down POLR3G in T24 and BIU87 cells. Three different sequences of siRNAs targeting POLR3G were designed, and the knockdown efficiency was validated by qPCR (Figure S1). The siRNA with the highest interfering efficiency was chosen to construct recombinant deficient lentivirus to knockdown POLR3G in T24 and BIU87 cells (Fig. 5A–C). Subsequently, we conducted CCK-8 assays to evaluate the impact of POLR3G on the viability of T24 and BIU87 cells. The results in both cell lines demonstrated a decrease in viability in POLR3G knockdown cells (Fig. 5D, E). Furthermore, we aimed to elucidate the impact of POLR3G on bladder cancer cell migration and invasion using Transwell migration and invasion assays. The results revealed reductions in the number of migrating cells and invading bladder cancer cells in the POLR3G knockdown groups compared to the corresponding control groups (Fig. 5F-I). Collectively, these in vitro results strongly suggest that targeting POLR3G might suppress the malignant phenotype of bladder cancer cells.

### RNA sequencing of T24 and BIU87 cells to investigate the function of POLR3G

**Fig. 6:**
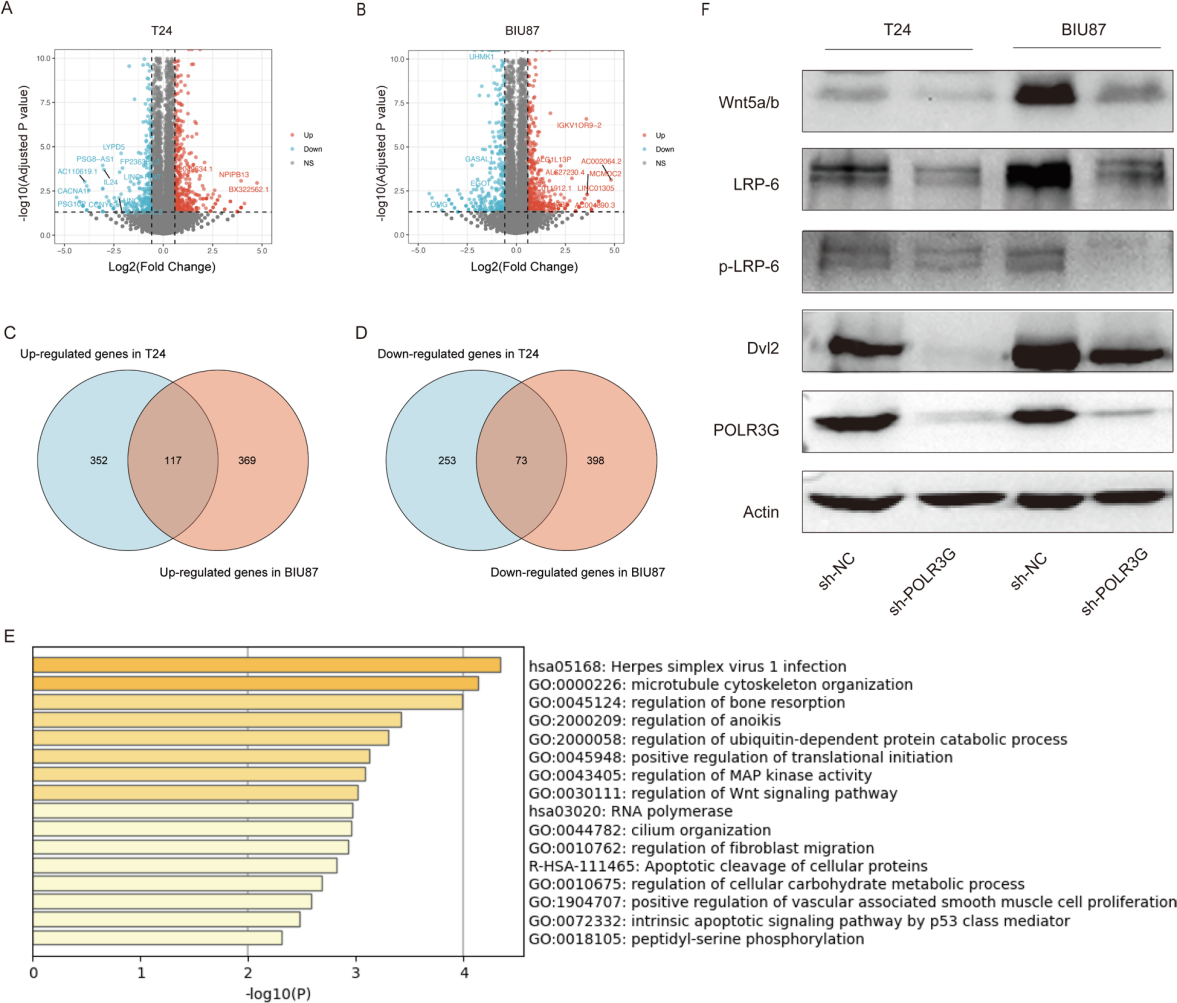
The transcriptomic analysis of bladder cancer cells upon POLR3G knockdown

To investigate the role of POLR3G in bladder cancer cells, we conducted RNA sequencing on POLR3G-knockdown and control bladder cancer cells. Through analysis of the sequencing data, we identified 469 up-regulated DEGs and 326 down-regulated DEGs in T24 cells (Fig. 6A), and 486 up-regulated DEGs and 471 down-regulated DEGs in BIU87 cells (Fig. 6B). A total of 117 DEGs were up-regulated in both T24 cells and BIU87 cells (Fig. 6C), and 73 DEGs were down-regulated in both T24 cells and BIU87 cells (Fig. 6D). Metascape was employed to perform the functional enrichment analysis of the DEGs (Fig. 6E) and the results showed that POLR3G was primarily involved in the RNA polymerase, and Apoptotic cleavage of cellular proteins signal pathways. Regarding molecular functions, POLR3G showed significant associations with regulation of Wnt signaling pathway and regulation of MAP kinase activity, regulation of ubiquitin-dependent protein catabolic process, positive regulation of translational initiation, regulation of fibroblast migration, regulation of cellular carbohydrate metabolic process, positive regulation of vascular associated smooth muscle cell proliferation, intrinsic apoptotic signaling pathway by p53 class mediator, and peptidyl-serine phosphorylation. To further confirm that POLR3G affected Wnt signaling pathways, we examined the Wnt signaling pathway-related gene expression. The results showed that the expression of Wnt 5a/b, Dvl2, LRP-6, and p-LRP-6 was markedly decreased when POLR3G was knocked down, which indicates that the activity of Wnt pathway was inhibited (Fig. 6F).

## Discussion

Bladder cancer is one of the most prevalent malignancies worldwide, with a significant impact on morbidity and mortality [1]. The prognosis of bladder cancer is influenced by a complex interplay of pathological, clinical, molecular, and other factors. Bladder tumors with larger size, higher grade, multifocality or lymphovascular invasion are at a higher risk of not responding to BCG treatment [16]. Higher stage and the presence of carcinoma in situ are associated with increased risk of disease progression and recurrence [4]. Additionally, patients with older age at diagnosis or poorer general health reported worse oncological outcomes [17]. The inflammatory and nutritional status can also influence the oncological outcomes. The modified Glasgow Prognostic Score based on C-reactive protein and albumin has been proved to be associated with the risk of recurrence of bladder cancer [18, 19]. Beyond pathological and clinical aspects, molecular biomarkers are increasingly recognized for their prognostic value. RNA-seq data from TCGA identified five expression subtypes of bladder cancer: luminal-papillary, luminal-infiltrated, luminal, basal-squamous, and neuronal. These subtypes showed distinct expression patterns of urothelial differentiation markers, p53 status, and immune markers. This classification system provides insights into bladder cancer heterogeneity and its impact on clinical outcomes, guiding personalized treatment approaches [20].

Over the past decade, ICIs and ADCs have revolutionized the treatment landscape of bladder cancer. The use of ICIs has not only shown promising results in the treatment of advanced bladder cancer [8–10], but also in neoadjuvant therapy settings [5, 21]. PURE-01trial reported a pathological complete response of 42% after neoadjuvant immunotherapy with pembrolizumab [21]. The combination of ADCs and ICIs has even challenged the first-line treatment position of platinum-based chemotherapy in advanced bladder cancer based on the results of EV-302/KEYNOTE-A39 trial [22]. Despite advances in diagnostic and therapeutic strategies, the prognosis for advanced bladder cancer remains poor, necessitating further research into its molecular mechanisms and potential therapeutic targets.

In previous study [13], we found POLR3G was up-regulated in bladder cancer, and high POLR3G expression was associated with higher tumor stage, tumor grade and other adverse clinicopathologic features. KM survival analysis showed that POLR3G expression was negatively associated with progression-free survival and disease specific survival as well as overall survival in bladder cancer patients. Thus, POLR3G might play an important role in promoting the development and progression of bladder cancer, and may serve as a novel therapeutic target.

POLR3G, a subunit of RNA polymerase III, is integral to the function of RNA polymerase III, which is responsible for transcribing small RNA molecules that are vital for protein synthesis and other cellular processes [23]. Disruption of POLR3G function leads to defects in RNA synthesis, which can have broad implications for cellular metabolism and growth [24]. Emerging evidence suggests that POLR3G is also involved in the regulation of stem cell pluripotency and differentiation [25, 26]. Research has shown that POLR3G is highly expressed in embryonic stem cells and is down-regulated upon differentiation [25]. This expression pattern indicates that POLR3G may play a role in maintaining the undifferentiated state of stem cells. The role of POLR3G in cancer has also garnered significant interest. Several studies have identified overexpression of POLR3G in various cancers, including prostate cancer and breast cancer [27, 28]. These findings show that downregulation of POLR3G impairs tumor growth, indicating the potential of POLR3G in cancer treatment.

In this study, we established a rat model of bladder cancer induced by N-butyl-N-(4-hydroxybutyl) nitrosamine (BBN) to investigate the dynamic changes in POLR3G protein expression during bladder cancer initiation and progression. Ultrasound imaging and pathological examinations were conducted at various stages to characterize the imaging features and urothelial pathology. Additionally, we performed in vitro functional assays to explore the impact of POLR3G on bladder cancer cell behavior. Through RNA sequencing and bioinformatics analysis of POLR3G knockdown cells, we identified potential molecular mechanisms underlying its role in bladder cancer, which were further validated by molecular experiments. The results of our study provide significant insights into the molecular mechanisms and signaling pathways involved in bladder cancer progression.

BBN-induced urothelial tumors in rodents resembled human urothelial lesions in their morphological and genetic characteristics [29, 30]. Microscopic lesions in rats usually start as simple hyperplasia, evolving into papillary and nodular hyperplasia, papilloma, and non-invasive carcinomas [29]. BBN tumors showed overexpression of markers of basal cancer subtype, and had a high mutation burden with frequent Trp53 (80%), Kmt2d (70%), and Kmt2c (90%) mutations by exome sequencing, similar to human MIBC [30]. Thus, BBN tumors have been proposed as a useful model for the study of urinary bladder carcinogenesis, as well as for evaluating new therapeutic strategies. The upregulation of POLR3G observed in our BBN-induced rat model of bladder cancer suggests that POLR3G plays a crucial role in the carcinogenesis and progression of bladder cancer. The positive correlation between POLR3G expression and the histopathological malignancy grade indicates that POLR3G could serve as a potential biomarker for bladder cancer progression.

Our functional experiments further elucidated the role of POLR3G in bladder cancer cell behavior. The knockdown of POLR3G significantly inhibited the proliferation, migration, and invasion capabilities of bladder cancer cells, highlighting its importance in tumor aggressiveness. The transcriptomic analysis revealed substantial changes in gene expression profiles upon POLR3G knockdown. The consistent upregulation of 117 genes and downregulation of 73 genes across both T24 and BIU87 cell lines suggested a robust and conserved regulatory role of POLR3G in bladder cancer. Furthermore, the enrichment of DEGs in the RNA polymerase and apoptotic cleavage of cellular proteins pathways, as well as their involvement in the Wnt and MAPK signaling pathways, underscored the complex regulatory networks that POLR3G may influence. These findings suggest that targeting POLR3G and its associated pathways could offer new therapeutic strategies for bladder cancer treatment, and enhance our understanding of the genetic and cellular mechanisms underlying bladder cancer progression and provide a foundation for future research aimed at elucidating the specific functions of these DEGs in bladder cancer.

The Wnt signaling pathway is a complex network of proteins that plays a crucial role in regulating cell growth, migration, and differentiation, and dysregulation of this pathway has been implicated in various cancers [31–33]. In bladder cancer, aberrant activation of the Wnt pathway has been observed, contributing to uncontrolled cellular proliferation and resistance to apoptosis [34]. The downregulation of Wnt pathway-related proteins such as Wnt5a/b, DVL2, LRP-6, and phosphorylated LRP-6 upon POLR3G knockdown was further confirmed by Western blotting, indicating that POLR3G may affect bladder cancer behavior through the Wnt signaling pathway. Our previous study found POLR3G may have significant implications for immune mechanisms in bladder cancer. More specifically, the expression of POLR3G was significantly correlated with the infiltrating levels of immune cells and the expression of immune checkpoint molecules in bladder cancer [13]. Given the known roles of the Wnt pathway in immune cell regulation and tumor immune evasion, our findings imply that POLR3G could influence the tumor microenvironment and immune surveillance in bladder cancer. This potential immunomodulatory role of POLR3G opens new avenues for research into immune-based therapies for bladder cancer, particularly those targeting the Wnt signaling pathway.

One limitation of this study is the reliance on the BBN-induced rat model to simulate bladder cancer progression. While this model is well-established and provides valuable insights into the disease's pathophysiology, it may not fully recapitulate the complexity of human bladder cancer. Additionally, the study's in vitro experiments, although informative, may not entirely reflect the in vivo tumor microenvironment, potentially limiting the generalizability of the findings. Furthermore, the study primarily focuses on the role of POLR3G, and while the results are compelling, other molecular players and pathways involved in bladder cancer progression may have been overlooked. Future studies should aim to validate these findings in human clinical samples and explore the interplay between POLR3G and other oncogenic pathways to provide a more comprehensive understanding of bladder cancer biology.

## Conclusions

In conclusion, our study elucidates the dynamic expression of POLR3G during bladder cancer progression and its significant role in modulating bladder cancer cell proliferation, migration, and invasion. The upregulation of POLR3G correlates with increased malignancy, and its knockdown results in substantial alterations in gene expression, particularly affecting the Wnt signaling pathways. These findings suggest that POLR3G is a potential biomarker and therapeutic target in bladder cancer. However, further research is warranted to validate these results in clinical settings and to explore the therapeutic potential of targeting POLR3G in combination with other molecular interventions. This study provides a foundation for future investigations into the molecular mechanisms underlying bladder cancer and highlights the importance of POLR3G in its pathogenesis.

## Data Availability

The RNA-seq data of this study have been deposited in the Galaxy (https://usegalaxy.org/u/xianhui_liu/h/rna-seq).
